# Opportunistic computed tomography to identify osteoporosis in primary hyperparathyroidism using sestamibi SPECT/CT scans

**DOI:** 10.1210/jendso/bvag246

**Published:** 2026-10-19

**Authors:** Gary Ka Wai Chan, Xi Xiong, Yan Ho Hui, Matrix Man Him Fung, David T W Lui

**Affiliations:** Nuclear Medicine Unit, Queen Mary Hospital, Hong Kong SAR, China; Department of Non-communicable Disease Epidemiology, Faculty of Epidemiology and Population Health, London School of Hygiene & Tropical Medicine, London WC1E 7HT, UK; Laboratory of Data Discovery for Health (D24H), Hong Kong Science and Technology Park, Hong Kong SAR, China; Centre for Safe Medication Practice and Research, Department of Pharmacology and Pharmacy, Li Ka Shing Faculty of Medicine, The University of Hong Kong, Hong Kong SAR, China; Nuclear Medicine Unit, Queen Mary Hospital, Hong Kong SAR, China; Department of Surgery, School of Clinical Medicine, Li Ka Shing Faculty of Medicine, The University of Hong Kong, Hong Kong SAR, China; Department of Medicine, School of Clinical Medicine, Li Ka Shing Faculty of Medicine, The University of Hong Kong, Hong Kong SAR, Hong Kong

**Keywords:** osteoporosis, primary hyperparathyroidism, sestamibi scan, CT, prediction

## Abstract

**Context:**

Osteoporosis is a well-recognized complication of primary hyperparathyroidism (PHPT).

**Objective:**

We aimed to develop an osteoporosis prediction model for PHPT patients using opportunistic computed tomography (CT)-derived Hounsfield unit (HU) measurements from sestamibi SPECT/CT.

**Methods:**

Sestamibi SPECT/CT images from 81 PHPT patients (60 with and 21 without osteoporosis diagnosed by dual-energy x-ray absorptiometry) were analyzed retrospectively. HUs of vertebral bodies were measured. Receiver operating characteristic (ROC) analysis was performed to determine the optimal HU cutoff for each vertebra in predicting osteoporosis. Multivariable logistic regression was used to identify independent clinical parameters associated with osteoporosis. A prediction model based on clinical parameters alone was built. Another prediction model was constructed by further incorporating the HU of the vertebra. Model performance improvement was assessed.

**Results:**

Age and body mass index (BMI) were independent risk factors for osteoporosis. The clinical prediction model (age and BMI) achieved an area under the receiver operating characteristic curve (AUROC) of 0.874. Incorporation of HU of T4 vertebra into the clinical model showed the best performance with AUROC of 0.966, sensitivity of 98.2%, and specificity of 85.7%, representing a significant improvement in prediction with change in C-statistics of 0.092 (95% CI, 0.021-0.163; *P* = .011), net reclassification index of 0.346 (0.041-0.650; *P* = .026), and incremental discrimination index of 0.329 (0.116-0.542; *P* = .003). After bootstrap internal validation, the optimism-corrected AUROC was 0.949 (0.882-1.000).

**Conclusion:**

Our study demonstrated the feasibility of integrating vertebral HU measurements from sestamibi SPECT/CT into a clinical prediction model for accurately identifying osteoporosis in PHPT patients, offering a potential tool for efficient bone health management and prioritizing timely surgical interventions.

Primary hyperparathyroidism (PHPT) is a recognized risk factor for osteoporosis and fractures due to its effect on bone metabolism and remodeling (1). Excessive secretion of parathyroid hormone (PTH) in patients with PHPT accelerates bone resorption, particularly affecting cortical bone, resulting in susceptibility to fragility fractures (2). PHPT is associated with a reduction in bone mineral density (BMD), especially in the cortical bone (2, 3). Early detection and management are crucial as parathyroidectomy can effectively halt and even reverse PHPT-related osteoporosis and reduce PHPT-related fractures (4, 5). PHPT-related osteoporosis is, therefore, a well-established indication for early parathyroidectomy (6), and all patients with PHPT are recommended to receive a 3-site dual-energy x-ray absorptiometry (DXA) scan for BMD assessment, that is, over the lumbar spine, hip, and distal radius (7, 8). Nonetheless, the waiting time for 3-site DXA is often prolonged (which could be up to years) in the public health care system due to the high demand for other disease conditions in an aging population (9). The delay in DXA for PHPT patients may lead to delayed diagnosis of osteoporosis, and hence delayed decision for surgery. An accurate and efficient prediction model for osteoporosis without the need for DXA in patients with PHPT helps triage resources for complication management and allows timely surgery for those most in need.

Sestamibi (MIBI) scan is often used to localize the diseased parathyroid gland(s) to facilitate minimally invasive parathyroidectomy. As recommended by international guidelines, adding SPECT/CT to MIBI scans improves the precision of localization and aids the surgical plan with high-resolution computed tomography (CT) images (10, 11). SPECT/CT is a widely used technique with modern gamma cameras. The value of opportunistic measurement of the Hounsfield unit (HU) from CT scans of other body parts to predict osteoporosis has been reported outside the context of PHPT (12, 13). This method allows for incidental BMD assessment by leveraging existing CT images without additional radiation exposure and saves imaging costs (14). SPECT/CT in MIBI scan thus represents an opportunity to predict osteoporosis in the same session of the scan, saving time during the patient journey.

Developing a prediction model incorporating HU of vertebrae from SPECT/CT of MIBI scan could help speed up the evaluation process for patients with PHPT for target organ involvements and timely management of the population with the highest fracture risk. Hence, we designed the present study to develop a prediction model for osteoporosis in patients with PHPT, incorporating the parameters derived from the opportunistic CT-derived HU measurements.

## Materials and methods

This study was approved by the institutional review board of the University of Hong Kong/Hospital Authority Hong Kong West Cluster (UW 24-225). Written informed consent from the participants was waived.

### Study design and population

We carried out a retrospective cohort study that included consecutive patients aged 50 years or older (and postmenopausal for women) with PHPT referred to our center for parathyroid MIBI scintigraphy performed between January 1, 2017 and June 30, 2024. Inclusion was restricted to patients aged 50 years or older (and postmenopausal for women) to ensure a cohort for whom a diagnosis of osteoporosis could be reliably established using standard T-score densitometric criteria via DXA, which is not validated for younger populations (15). Inclusion criteria required MIBI SPECT/CT imaging for the parathyroid scan, and DXA scan performed within 12 months of MIBI SPECT/CT. The diagnosis of PHPT was reviewed from the electronic health records and adjudicated by an endocrinologist (D.T.W.L.) and an endocrine surgeon (M.M.H.F.) on the investigator team. Demographic and anthropometric data and laboratory parameters were retrieved from the electronic health records.

### Acquisition and analysis of computed tomography images

SPECT/CT images from the parathyroid scans were obtained from the local picture archiving and communication system (PACS). SPECT/CT examinations were performed using the General Electronic Discovery 670 DR system. The CT component of the SPECT/CT scan was acquired using a helical scan with a pitch of 1.35, 120 kV, and an automatic mA setting with a noise index of 13.16. Examinations were performed without the administration of contrast media. The CT images were reconstructed into 1.25-mm thick axial slices. A typical MIBI SPECT/CT in our center covers the vertebral bodies from C1 to T4.

The CT images were reviewed using the General Electronic Xeleris 4 DR software with the Q.Volumetrix MI module. Attenuation measurements were performed by a nuclear medicine physician (G.K.W.C.) with 4 years of experience. For each vertebra (C3-T4), a single axial CT slice located at mid-height of the vertebral body (equidistant from the superior and inferior end plates) was selected. Click-and-drag circular regions of interest (ROIs) were placed in the medullary space of the vertebral bodies on this central axial slice. Areas of cortical margin, heterogeneous density, venous plexus, or implants were avoided when placing ROIs. Fractured or severely degenerated vertebrae, vertebrae with large osseous lesions, and vertebrae that were entirely or partially out of field of view were excluded. The numbers of excluded vertebrae were as follows: C3 (n = 1), C4 (n = 2), C5 (n = 5), C6 (n = 2), T3 (n = 2), and T4 (n = 3). The mean values of HU of the ROIs were taken as the CT attenuation values of the respective vertebrae.

### Acquisition and analysis of dual-energy x-ray absorptiometry

DXA results were retrieved from PACS, including T-scores at the lumbar spine (L1-L4), femoral neck, total hip, and distal one-third of radius. DXA scans were performed using a Hologic Horizon A (S/N 201168) according to standard acquisition and analysis techniques recommended by the International Society for Clinical Densitometry (15). The least significant change of the DXA in our center was 0.030 g/cm^2^ over L1 to L4 and 0.026 g/cm^2^ over the total hip. The lowest T-scores among the lumbar spine, femoral neck, total hip, and distal one-third of the radius were used to classify patients into normal, osteopenia, or osteoporosis categories based on World Health Organization criteria (16). The presence of osteoporosis was defined by the lowest T-score less than or equal to −2.5.

### Statistical analyses

Data were analyzed using SPSS version 30 and Stata version 16. A *P* value less than .05 was considered statistically significant for all analyses. Data were described as mean with SD. Between-group comparisons were performed using *t* test and chi-square test as appropriate.

Multivariable logistic regression analyses were used to determine the independent factors associated with osteoporosis. Factors with statistically significant association with osteoporosis in the univariate analyses were selected in the multivariable models. A clinical prediction model for osteoporosis was developed based on these clinical parameters alone. Subsequently, MIBI SPECT/CT-derived vertebral HU measurements (as a dichotomous variable at its optimal cutoff) were added to the clinical prediction model. Receiver operating characteristic (ROC) analyses were conducted for the clinical prediction model and the model incorporating vertebral body Hounsfield units (HU) from C3 to T4 derived from SPECT/CT. Optimal cutoffs for each vertebra were determined using Youden’s index. These prediction models were compared using the likelihood ratio test, integrated discrimination improvement (IDI), and net reclassification improvement (NRI) indices. We used bootstrapping with 200 replications to calculate 95% CIs.

To assess internal validity, we used bootstrapping to quantify optimism in model performance. The entire modeling process, including the recalculation of the optimal cutoff for vertebrae, was repeated across 200 bootstrapped samples. The individual area under the receiver operating characteristic curve (AUROC) values from these samples were averaged to derive an optimism-corrected AUROC.

### Sensitivity analyses

Sensitivity analyses were performed to demonstrate the robustness of the results. First, analyses were repeated after the exclusion of individuals without BMD measurements over the distal radius. Second, analyses were repeated by the inclusion of the C7 vertebra (with complete data in our study and commonly covered in most MIBI SPECT/CT scans) instead of the T4 vertebra used in the main analysis. Third, since there were 11 individuals who were on antiosteoporosis treatment (alendronate, n = 8; denosumab, n = 3) between MIBI and DXA scans, analyses were repeated after excluding these 11 individuals. Finally, to assess predictive performance across skeletal sites, site-specific analyses were conducted by defining osteoporosis separately at the lumbar spine, either the total hip or femoral neck, and the distal one-third radius using a T-score less than or equal to −2.5. For each outcome, the optimal T4 HU cutoff was determined separately by maximizing Youden’s index, and a prediction model incorporating age, body mass index (BMI), and dichotomized T4 HU was fitted.

## Results

A total of 81 patients were included. Sixty (74%) were classified as having osteoporosis by DXA. Nine patients did not have DXA performed over the distal radius. The median interval between MIBI SPECT/CT and DXA was 193 days; 18 patients (22.2%) had DXA performed within 3 months and a cumulative total of 36 patients (44.4%) had DXA performed within 6 months. During the interval between imaging studies, 9 patients (11.1%) underwent parathyroidectomy, with a median duration between surgery and DXA of 54 days (interquartile range, 19.5-111 days). The clinical characteristics of the cohort are summarized in Table 1. Patients with osteoporosis were older and had lower BMI and higher PTH levels than those without osteoporosis. There was no statistically significant difference in sex preponderance, smoking or alcohol consumption habits, adjusted calcium levels, estimated glomerular filtration rates, total vitamin D level, comorbidities (diabetes and hyperthyroidism), and history of fracture. The optimal cutoffs of the C3 to T4 vertebrae were obtained and are listed in Table 2.

**Table 1:** Clinical characteristics of patients with primary hyperparathyroidism, categorized according to the presence of osteoporosis

	Patients without osteoporosis	Patients with osteoporosis	*P*
No.	21	60	—
Age, y	63.0 ± 9.8	71.0 ± 10.5	.003
Female, %	15 (71.4%)	48 (80.0%)	.416
BMI	26.7 ± 3.1	23.1 ± 3.9	<.001
Active smoker	0 (0.0%)	0 (0.0%)	
Active drinker	1 (4.8%)	3 (5.0%)	≥.999
PTH, pmol/L	9.1 ± 5.4	12.5 ± 8.0	.039
ALP, U/L	63.0 ± 36.7	88.5 ± 37.4	.660
Adjusted calcium, mmol/L	2.70 ± 0.14	2.72 ± 0.17	.518
eGFR, mL/min/1.73m^2^	68.4 ± 24.0	71.5 ± 20.1	.597
Total vitamin D, nmol/L	65.7 ± 23.6	80.2 ± 22.1	.797
Diabetes mellitus	3 (14.3%)	16 (26.7%)	.372
Hyperthyroidism	0 (0.0%)	0 (0.0%)	
History of fracture	3 (14.3%)	9 (15%)	.937
Densitometric diagnosis			<.001
Normal	4 (19.0%)	0 (0%)	
Osteopenia	17 (81.0%)	0 (0%)	
Osteoporosis	0 (0%)	60 (100%)	
BMD T-score			
Lumbar spine	−0.98 ± 1.10	−2.64 ± 1.44	<.001
Femoral neck	−1.40 ± 0.75	−2.79 ± 0.73	<.001
Total hip	−0.54 ± 0.77	−2.12 ± 0.60	<.001
Distal radius	−0.75 ± 1.14	−3.47 ± 1.39	<.001

Abbreviations: ALP, alkaline phosphatase; BMD, bone mineral density; BMI, body mass index; eGFR, estimated glomerular filtration rate; PTH, parathyroid hormone.

**Table 2:** Performance of prediction models for osteoporosis on incorporation of individual vertebrae along C3 to T4

Vertebral level	No. of patients with complete data	Threshold CT attenuation, HU	AUROC (95% CI)*^a^*	Cutoff value*^b^*	Sensitivity, %	Specificity, %	Positive predictive value, %	Negative predictive value, %
C3 axial	80	311.0	0.892 (0.815-0.970)	0.747	88.3	75.0	91.4	68.2
C4 axial	79	315.0	0.909 (0.842-0.976)	0.730	86.7	78.9	92.9	65.2
C5 axial	76	316.0	0.915 (0.857-0.973)	0.893	67.9	100.0	100.0	52.6
C6 axial	79	234.0	0.906 (0.830-0.983)	0.733	84.5	85.7	94.2	66.7
C7 axial	81	227.0	0.890 (0.815-0.964)	0.786	76.7	85.7	93.9	56.3
T1 axial	81	197.0	0.894 (0.824-0.963)	0.832	76.7	90.5	95.8	57.6
T2 axial	81	181.0	0.890 (0.813-0.967)	0.591	91.7	76.2	91.7	76.2
T3 axial	79	182.0	0.916 (0.837-0.995)	0.599	93.2	80.0	93.2	80.0
T4 axial	78	174.0	0.966 (0.915-1.000)	0.335	98.2	85.7	94.9	94.7

Abbreviations: AUROC, area under receiver operating characteristic curve; CT, computed tomography.

^*a*^The AUROC reported in this table represents the AUROC of the prediction model incorporating age, body mass index, and the Hounsfield unit of the respective vertebra indicated in that row.

^*b*^The diagnostic cutoff value for the model on osteoporosis was determined based on the optimal point with the maximum Youden’s index on the ROC curve.

### Clinical prediction model for osteoporosis

Multivariable logistic regression analysis showed that only age (adjusted odds ratio [OR] 1.107; 95% CI, 1.034-1.185; *P* = .003) and BMI (adjusted OR 0.684; 95% CI, 0.555-0.843; *P* < .001) were the independent risk factors for osteoporosis. A clinical prediction model incorporating age and BMI was built, represented by the formula log(*P*/(1 − *P*)) = 3.59 + 0.10 × age (year) – 0.38 × BMI, where *P* is the probability of osteoporosis. The AUROC of this clinical prediction model was 0.874 (95% CI, 0.786-0.961). The optimal cutoff was 0.707, resulting in a sensitivity of 82.5%, specificity of 85.7%, positive predictive value (PPV) of 94.0%, and negative predictive value (NPV) of 64.3%.

### Prediction model incorporating sestamibi SPECT/CT vertebral Hounsfield units

We evaluated the predictive ability of models incorporating the CT attenuation of vertebrae on MIBI scans (C3-T4). As the CT attenuation of C3 to T4 correlated highly with each other, we evaluated the predictive ability of models by adding one vertebra at a time to the clinical prediction model (see Table 2 and Fig. 1). The AUROC ranged from 0.890 (C7 or T2 vertebra) to 0.966 (T4 vertebra). Overall, the AUROC was higher after incorporating the CT attenuation of vertebrae when compared to the clinical prediction model.

**Figure 1:**
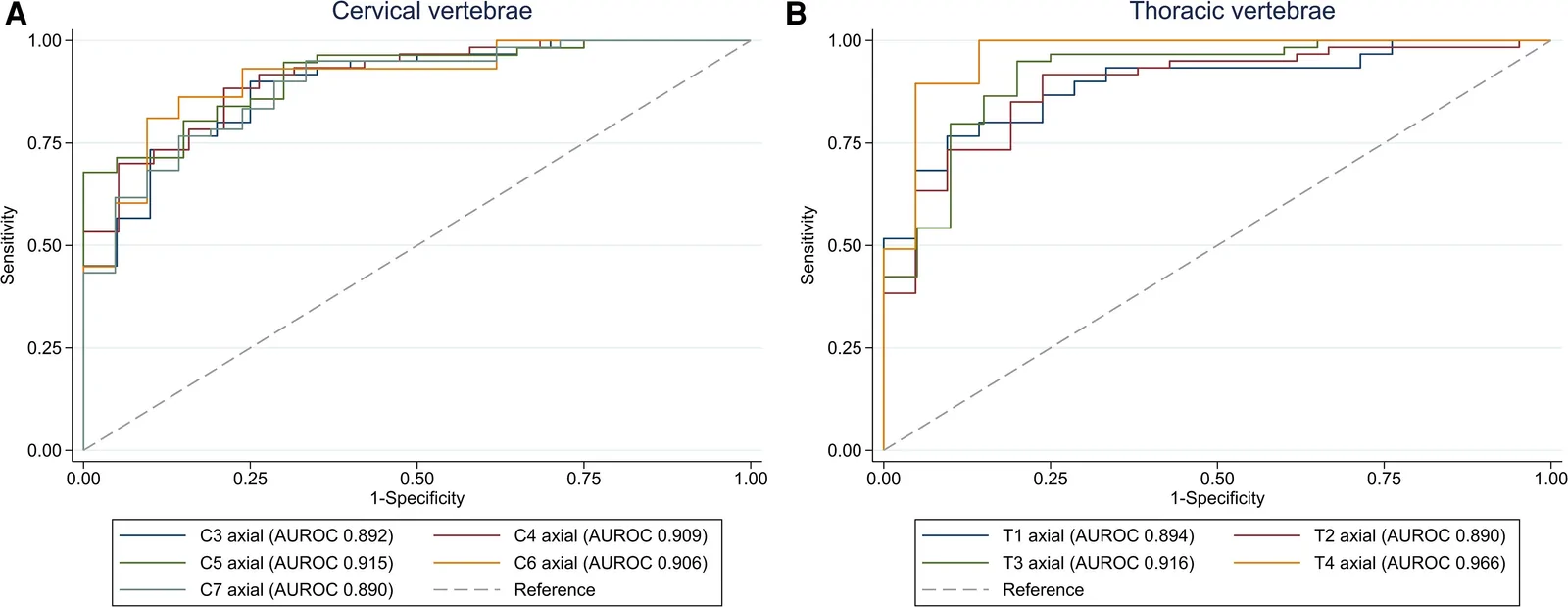
Receiver operating characteristic curves of prediction models incorporating vertebral Hounsfield unit measurements from C3 to T4. The AUROCs shown in this figure represent the performance of clinical models incorporating age, body mass index, and the Hounsfield unit measurement of the corresponding vertebra in A, cervical spine and B, thoracic spine, respectively, as reported in Table 2. AUROC, area under receiver operating characteristic curve.

Of all the prediction models, the model incorporating the CT attenuation of the T4 vertebra showed the best AUROC at 0.966 (95% CI, 0.915-1.000) (Table 3) represented by the formula log(*P*/(1 − *P*)) = 6.89 – 4.82 × T4 axial + 0.10 × age (year) – 0.37 × BMI, where *P* is the probability of osteoporosis and “T4 axial” is a binary factor (0 if T4 attenuation is below the optimal cutoff and 1 if it is at or above the cutoff). At the optimal cutoff of 0.335, sensitivity was 98.2%, specificity was 85.7%, PPV was 94.9%, and NPV was 94.7%. With the addition of T4 vertebra to the risk prediction model, AUROC increased from 0.874 to 0.966 (ΔAUROC 0.092 [95% CI, 0.021-0.163]; *P* = .011; Fig. 2). This combined model showed statistically significant improvement compared with the clinical prediction model using different metrics: The change in likelihood was 29.5 (*P* < .001), NRI was 0.346 (95% CI, 0.041-0.650; *P* = .026), and IDI was 0.329 (95% CI, 0.116-0.542; *P* = .003). After bootstrap internal validation, the optimism-corrected AUROC was 0.949 (95% CI, 0.882-1.000), indicating excellent discrimination.

**Figure 2:**
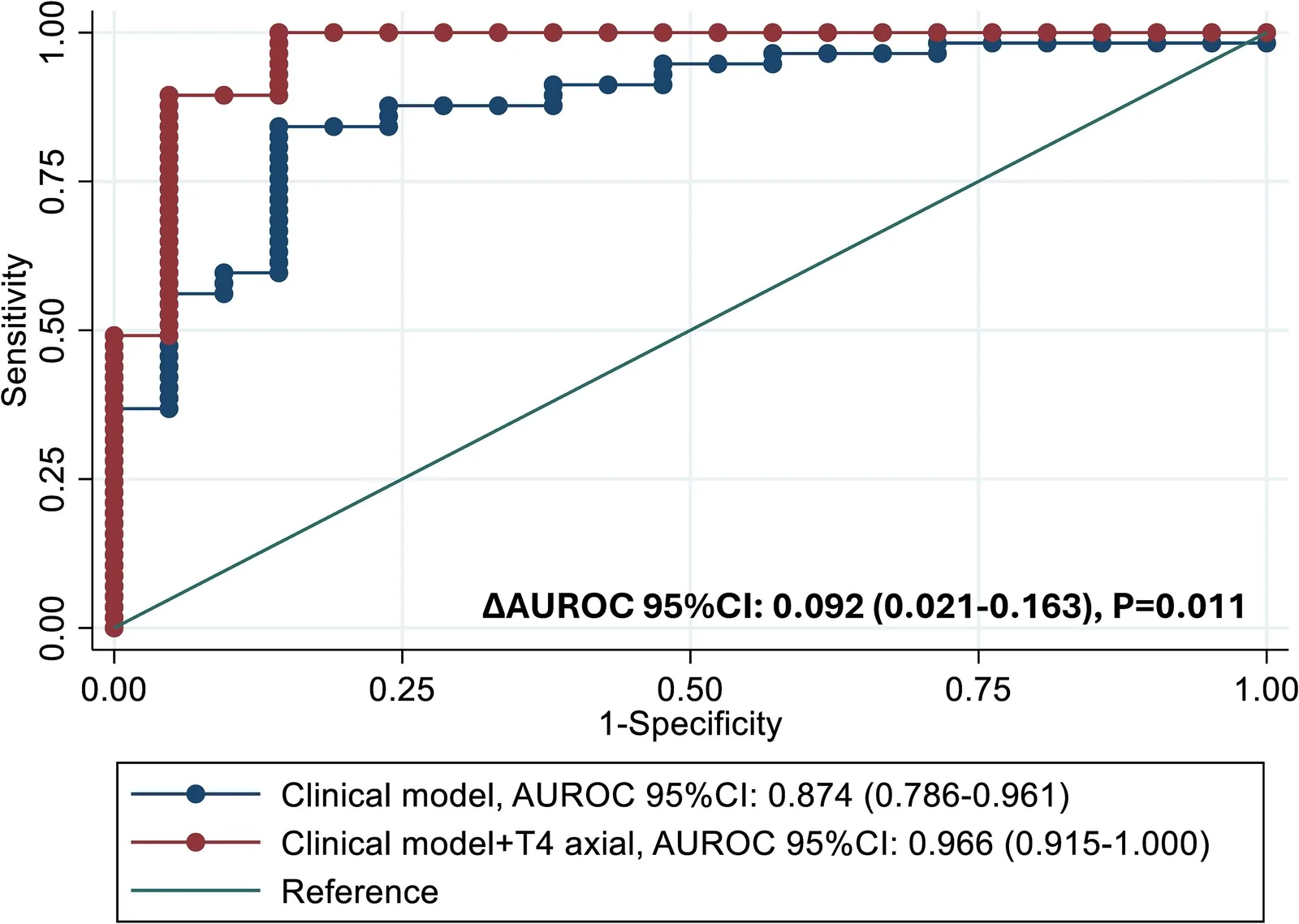
The incremental predictive value by incorporating attenuation value of T4 vertebra to the clinical prediction model. AUROC, area under receiver operating characteristic curve.

**Table 3:** Performance of clinical model and the prediction model incorporating T4 vertebral attenuation value

	Clinical model*^a^*	Clinical model + T4 axial*^a^*
Cutoff value*^b^*	0.707	0.335
AUROC	0.874 (95% CI, 0.786-0.961)	0.966 (95% CI, 0.915-1.000)
Sensitivity, %	82.5	98.2
Specificity, %	85.7	85.7
PPV, %	94.0	94.9
NPV, %	64.3	94.7

Abbreviations: AUROC, area under receiver operating characteristic curve; NPV, negative predictive value; PPV, positive predictive value.

^*a*^Due to missing values in T4 attenuation measurements in 3 patients, the clinical model and the combined model were developed using complete cases, resulting in a sample of 78 patients.

^*b*^The diagnostic cutoff value for the model on osteoporosis was determined based on the optimal point with the maximum Youden’s index on the ROC curve.

### Sensitivity analyses

In the first sensitivity analysis excluding the 9 patients without distal radius BMD, baseline clinical characteristics were compared between patients with (n = 72) and without (n = 9) distal radius DXA scans, showing no statistically significant differences in age, sex, BMI, drinking status, PTH, adjusted calcium, renal function, total vitamin D level, diabetic status, and history of fracture (*P* > .05 for all), although baseline alkaline phosphatase (ALP) was higher in those without distal radius DXA (118.7 ± 40.4 vs 86.0 ± 35.2 IU/L; *P* = .012). The equation for the prediction of osteoporosis using clinical parameters was log(*P*/(1 − *P*))= 5.84 + 0.09 × age (year) – 0.42 × BMI, where *P* is the probability of osteoporosis. This model showed an AUROC of 0.883 (95% CI, 0.801-0.966). The combined model incorporating HU of T4 vertebra was represented by the equation log(*P*/(1 − *P*)) = 12.06 – 4.95 × T4 axial + 0.05 × age (year) – 0.45 × BMI, where *P* is the probability of osteoporosis and “T4 axial” is a binary factor (0 if T4 attenuation is below the optimal cutoff and 1 if it is at or above the cutoff). The combined model remained excellent, in line with the main analysis, with an AUROC of 0.965 (95% CI, 0.916-1.000). The increment in C-statistics was 0.082 (95% CI, 0.003-0.160; *P* = .041). The predictive ability was significantly improved when compared to the clinical model, with a change in likelihood ratio of 24.450 (*P* < .001), NRI of 0.464 (95% CI, 0.117-0.811; *P* = .009), and IDI of 0.324 (95% CI, 0.104-0.544; *P* = .004).

In the second sensitivity analysis, by replacing the CT attenuation of T4 with that of the C7 vertebra, the formula for the prediction was log(*P*/(1 − *P*)) = 5.86 – 2.30 × C7 axial + 0.07 × age (year) – 0.34 × BMI, where *P* is the probability of osteoporosis and “C7 axial” is a binary factor (0 if C7 attenuation is below the optimal cutoff and 1 if it is at or above the cutoff). With the addition of C7 vertebra to the risk prediction model, AUROC increased from 0.858 to 0.890 (ΔAUROC 0.032 [95% CI, −0.031 to 0.094]; *P* = .319). The AUROC of this prediction model was 0.890 (95% CI, 0.815-0.964) (see Table 2), significantly improving from the clinical model with a change in likelihood ratio of 10.430 (*P* = .001). There was a trend toward significance for NRI (0.205; 95% CI, −0.088 to 0.498; *P* = .171) and IDI (0.112; 95% CI, −0.035 to 0.259; *P* = .135).

In the third sensitivity analysis excluding the 11 patients receiving antiosteoporosis treatment, 70 patients remained in the analysis. Baseline comparison revealed that patients receiving antiosteoporosis therapy were significantly older (75.2 ± 10.4 vs 67.9 ± 10.7 years; *P* = .040), and had higher total vitamin D levels (90.9 ± 21.4 vs 73.2 ± 22.7 nmol/L; *P* = .026). There were no statistically significant differences in sex, BMI, drinking status, PTH, ALP, adjusted calcium, renal function, diabetic status, or history of fracture (*P* > .05). The formula for the prediction was log(*P*/(1 − *P*)) = 9.31 – 5.00 × T4 axial + 0.07 × age (year) – 0.40 × BMI, where *P* is the probability of osteoporosis and “T4 axial” is a binary factor (0 if T4 attenuation is below the optimal cutoff and 1 if it is at or above the cutoff). With the addition of T4 vertebra to the risk prediction model, AUROC increased from 0.852 to 0.964 (ΔAUROC 0.112 [95% CI, 0.031-0.192]; *P* = .007). The AUROC for this combined prediction model was 0.964 (95% CI, 0.909-1.000), which significantly improved from the clinical model with a change in likelihood ratio of 29.560 (*P* < .001) and IDI (0.361; 95% CI, 0.180-0.542; *P* < .001). There was a trend toward significance for NRI (0.290; 95% CI, −0.023 to 0.603; *P* = .069).

In the fourth sensitivity analysis, osteoporosis was present at the lumbar spine in 43 of 78 patients (55.1%), at either the total hip or femoral neck in 39 of 81 patients (48.1%), and at the distal one-third radius in 44 of 72 patients (61.1%). The site-specific prediction models incorporating age, BMI, and dichotomized T4 HU demonstrated good discrimination across all 3 skeletal sites. The AUROC was 0.877 (95% CI, 0.797-0.958) for lumbar spine osteoporosis, 0.882 (95% CI, 0.806-0.958) for total hip or femoral neck osteoporosis, and 0.868 (95% CI, 0.772-0.963) for distal one-third radius osteoporosis (Table 4).

**Table 4:** Site-specific prevalence of osteoporosis and performance of clinical prediction models incorporating T4 vertebral Hounsfield units

Osteoporosis site	No. of patients with complete data	Osteoporosis, n/N (%)*^a^*	AUROC (95% CI)*^b^*
Lumbar spine	75	43/78 (55.1%)	0.877 (0.797-0.958)
Total hip or femoral neck	78	39/81 (48.1%)	0.882 (0.806-0.958)
Distal one-third radius	69	44/72 (61.1%)	0.868 (0.772-0.963)

Abbreviation: AUROC, area under receiver operating characteristic curve.

^*a*^The denominator is the number of patients with an available T-score at the corresponding skeletal site.

^*b*^The AUROC represents the performance of site-specific prediction models incorporating age, body mass index, and dichotomized T4 HU. For each site-specific outcome, the optimal T4 HU cutoff was determined by maximizing Youden’s index.

## Discussion

Our study demonstrated the utility of opportunistic bone health assessment using MIBI SPECT/CT in patients with PHPT. By incorporating quantitative HU measurements from MIBI SPECT/CT scans, we developed a prediction model that outperformed a model using clinical parameters alone in identifying patients with osteoporosis defined by DXA. The results highlighted the potential adjunctive role of MIBI SPECT/CT scans for identifying osteoporosis in patients with PHPT to allow triage for timely surgery and bone health management.

Our study illustrates the application of the principle of “opportunistic CT” (17) in PHPT, a population at higher risk of osteoporosis and fractures. The key strength of such an approach is the opportunistic use of existing imaging data, which can provide additional diagnostic information without further radiation exposure or dedicated imaging examinations. This is particularly advantageous for patients with PHPT, who often undergo SPECT/CT for parathyroid localization as part of their clinical management. This approach has the potential to enable earlier referral for DXA scans or prompt initiation of appropriate treatment for osteoporosis, thereby reducing the risk of fragility fractures in this patient group. Existing studies have explored opportunistic CT imaging for bone health assessments in other patient populations, which were more heterogeneous in the fracture risk profiles. Most studies of opportunistic CT used the HU over lumbar vertebrae, most commonly L1 (18). Pickhardt and colleagues (14) identified a cutoff of 135 HU at the L1 vertebra on routine abdominal CT scans to identify individuals with osteoporosis in an American academic center. Li and colleagues (19) showed that this cutoff at the L1 vertebra was likely generalizable to the Chinese population. Apart from an unselected population, Jain and colleagues (20) showed that opportunistic CT using L1 vertebra could be applied to populations at higher fracture risk, such as a diabetes population, with modification of the cutoff. In contrast, fewer studies examined the utility of thoracic vertebrae on CT in the prediction of osteoporosis. A study in the Netherlands of patients with severe chronic obstructive pulmonary disease (a condition associated with accelerated bone loss) showed a strong correlation between the average attenuation of the 3 thoracic vertebrae (T4, T7, and T10) and the lowest BMD of the hip and lumbar spine (*r* = 0.827; *P* < .001). The attenuation threshold of 147 HU predicted the presence of osteoporosis with an AUROC of 0.969 (21). We extended the current understanding of opportunistic CT to multiple vertebrae from C3 to T4 commonly covered by SPECT/CT images of MIBI scans, showing a statistically significant improvement in the prediction of osteoporosis by including the attenuation values of these vertebrae. Our study illustrated a better prediction of osteoporosis when the attenuation values of more inferior vertebral levels were incorporated. A possible explanation is that degenerative changes are more common in the cervical spine, which increases HU. Interestingly, although PHPT is known to have a more significant effect on cortical bone than trabecular bone, our prediction model using attenuation values of the trabecular compartment of the vertebrae performed well in identifying patients with osteoporosis in PHPT. Cortical BMD assessment remains challenging (22). Cortical bone assessment can be performed using quantitative CT or dual-energy CT (22, 23). The use of opportunistic CT in assessing cortical bone is not widely studied. Studies evaluating the role of cortical thickness of the lumbar spine on opportunistic CT in predicting osteoporosis yielded suboptimal results with lower sensitivity and accuracy compared with HU of trabecular bones (24, 25).

The incorporation of CT-derived HU measurements significantly improved the prediction of osteoporosis with an AUROC of 0.966. Notably, the sensitivity and specificity were high, indicating excellent performance for opportunistic risk stratification in PHPT. This prediction model could help manage patients with PHPT in two ways. First, it enables the immediate identification of patients at high risk for skeletal fragility during routine localization studies, providing clinicians with early objective evidence of potential target organ involvement while formal densitometry is pending. Second, it helped triage patients with PHPT to prioritize those with a high likelihood of osteoporosis to receive a DXA scan to establish baseline bone health status for subsequent monitoring after curative treatment, streamlining the resource allocation in the heavily burdened public health-care systems. Importantly, these patients had added indications for timely surgery.

Notably, our study of the predictive ability of opportunistic CT for osteoporosis in this cohort of patients with PHPT considered both clinical parameters and attenuation values instead of attenuation values in isolation. The reasonable predictive ability of the clinical prediction model for osteoporosis in PHPT, as shown in our study (AUROC of 0.874), reiterated the importance of clinical risk factors in the prediction. Indeed, age and BMI form the backbones of many of the clinical risk prediction models of osteoporosis for the general population, such as FRAX (26) and the Osteoporosis Self-Assessment Tool for Asians (OSTA) (27).

Our study provides novel insights regarding the application of opportunistic CT to identify osteoporosis. Our findings are likely generalizable to other centers given the widespread use of MIBI SPECT/CT, which is the recommended standard for detecting hyperfunctioning parathyroid glands by the European Association of Nuclear Medicine (10). The use of HU from CT imaging, a universally applicable metric across scanners, enhances the reproducibility of our results. Nonetheless, our study results should be interpreted with certain limitations. First, given the study’s retrospective design, the SPECT/CT and DXA could not be performed simultaneously. By limiting the interval between SPECT/CT and DXA to 12 months, we minimized the potential change in the BMD that would influence the study results. Second, the sample size was relatively small. Third, possible selection bias cannot be excluded. Since our cohort was restricted to patients referred for MIBI SPECT/CT localization studies, which are typically ordered for individuals meeting surgical criteria or planning parathyroidectomy, our study population may not be fully representative of the broader PHPT population, particularly milder or asymptomatic cases managed conservatively without localization imaging. Fourth, all vertebral HU measurements were performed by a single nuclear medicine physician, and formal interobserver or intraobserver reproducibility was not evaluated. Finally, the proposed model has not yet been validated in a prospective cohort or any external data set and our study included only Chinese patients, pending external validation in other ethnicities.

### Conclusion

In patients with PHPT, the integration of vertebral body HU measurements from MIBI SPECT/CT with clinical parameters can accurately identify osteoporosis. The opportunistic use of CT imaging data from MIBI SPECT/CT may enable more efficient bone health management, and triage resources from timely surgery in patients with PHPT.

## Data Availability

Some or all datasets generated during and/or analyzed during the current study are not publicly available but are available from the corresponding authors on reasonable request.

## Abbreviations

ALP: alkaline phosphatase
AUROC: area under receiver operating characteristic curve
BMD: bone mineral density
BMI: body mass index
CT: computed tomography
DXA: dual-energy x-ray absorptiometry
HU: Hounsfield unit
IDI: integrated discrimination improvement
MIBI: sestamibi
NPV: negative predictive value
NRI: net reclassification improvement
PACS: picture archiving and communication system
PHPT: primary hyperparathyroidism
PPV: positive predictive value
PTH: parathyroid hormone
ROC: receiver operating characteristic
ROI: region of interest
